# A Decision Support System to Facilitate Identification of Musculoskeletal Impairments and Propose Recommendations Using Gait Analysis in Children With Cerebral Palsy

**DOI:** 10.3389/fbioe.2020.529415

**Published:** 2020-11-27

**Authors:** Kohleth Chia, Igor Fischer, Pam Thomason, H. Kerr Graham, Morgan Sangeux

**Affiliations:** ^1^Murdoch Children’s Research Institute, Melbourne, VIC, Australia; ^2^The Royal Children’s Hospital Melbourne, Parkville, VIC, Australia; ^3^Department of Paediatrics, University of Melbourne, Parkville, VIC, Australia

**Keywords:** decision support system, gait analysis, cerebral palsy, orthopaedics, random forest, peadiatrics

## Abstract

The identification of musculoskeletal impairments from gait analysis in children with cerebral palsy is a complex task, as is formulating (surgical) recommendations. In this paper, we present how we built a decision support system based on gait kinematics, anthropometrics, and physical examination data. The decision support system was trained to learn the association between these data and the list of impairments and recommendations formulated historically by experienced clinicians. Our aim was 2-fold, train a computational model that would be representative of data-based clinical reasoning in our center, and support new or junior clinicians by providing pre-processed impairments and recommendations with the associated supportive evidence. We present some of the challenges we faced, such as the issues of dimensionality reduction for kinematic data, missing data imputations, class imbalance and choosing an appropriate model evaluation metric. Most models, i.e., one model for each impairments and recommendations, achieved a weighted Brier score lower than 0.20, and sensitivity and specificity greater than 0.70 and 0.80, respectively. The results of the models are accessible through a web-based application which displays the probability predictions as well as the (up to) 5 best predictors.

## Introduction

Cerebral palsy (CP) refers to a group of disorders due to a brain lesion that occurred shortly before or after birth (Graham et al., 2016). CP is the most common cause of physical disability in children, with a prevalence of around 2.5 per 1000 births in developed countries (McIntyre, 2018). Due to the brain lesion, secondary musculoskeletal impairments often develop and worsen during childhood and adolescence. Clinical and physical examinations as well as three-dimensional gait analysis (3DGA) are utilized to determine how the musculoskeletal impairments affect the capacity of an individual to walk (Wren et al., 2011a, b).

Identifying neuro-musculoskeletal problems that impact the walking function of children with CP is a difficult process that involves multiple components. The diagnostic matrix (Davids et al., 2004) includes clinical history and diagnosis, classification and functional scales, physical examination, such as passive range of joint motion and muscle strength, and 3DGA which provides the kinematics and kinetics of the lower limb joints during walking as curves. In the latter analysis, the effects of the musculoskeletal impairments on gait may be detected from abnormal features present in the kinematic and kinetic curves. Features that might be interpreted include the magnitude and waveform of the different curves, the difference between the patient’s curve and those from healthy individuals or the differences between the left and right limb curves. The final surgical recommendations also incorporate diagnostic imaging and examination under anesthesia (Davids et al., 2004).

A clinician needs to invest significant time and energy into assimilating all the available clinical information to determine the impairments that affect a child’s capacity to walk. For example, 3DGA feature interpretation is not straightforward because: (i) one feature in one curve may relate to several impairments, (ii) there may be several features corresponding to several impairments, (iii) one impairment may lead to abnormal features in several other curves, and (iv) kinematics features may be primarily related to an impairment, compensatory or obligatory because of an impairment. Interpretation therefore requires analyzing the features observed simultaneously in the 24 kinematic and kinetic graphs in conjunction with results from the physical examinations and information from the other assessments.

We developed a decision support system to facilitate the process of identifying musculoskeletal impairments from the typical gait analysis assessments. Both the complexity of the identification process and the necessity to support this process using computerized tools has been recognized in the past, as early as the 1990s (Weintraub et al., 1990; Johnson et al., 1996; Simon, 2004), however, with limited uptake in practice by the clinical gait analysis community. Work to design decision support systems for gait analysis is continuing (Wagner et al., 2019). Machine learning concepts and algorithms are now applied to a range of tasks pertaining to gait analysis thanks to advancement in computation power and widespread use of databases to store clinical and gait analysis data. These tasks range from automated classification (Lai et al., 2009; Rozumalski and Schwartz, 2009; Sangeux et al., 2015) to predicting outcomes (Schwartz et al., 2013) to data-driven optimal clinical decision making (Ries et al., 2014).

The core of the decision support system we developed is a group of predictive models which use physical examination and kinematic data to identify impairments and surgical recommendations. The models were trained on a historical dataset to predict, based on the current clinical findings for the current child, what the impairment list and surgical recommendations would have been identified by the clinicians in the past. In other words, the predictive models are concerned about replicating the behaviors of clinicians in the past and may be viewed as an objective and probabilistic storage of clinical reasoning. Our objective was to develop models able to support a clinician by providing an answer to the question: “What my-past-self, or experienced predecessors in this center, would have decided based on similar data?” As such, these models may be particularly useful to clinicians with less clinical experience.

In our gait laboratory, the clinical decision-making process is separated into two components. Firstly, we identify the impairments, objectively from the clinical data. Secondly, management options which may include orthopaedic surgery are selected. We therefore developed this decision support system for both components. However, instead of being sequential, we developed these two decision support components independently of each other. The surgical recommendation system depends on the clinical data only not the (machine) identified impairments. The reason for making these independent is so that the quality of the surgical recommendation system will not be limited by the quality of the impairment identification system. Thus, even if the system fails to return a correct impairment list, there is still a possibility for the second component of the system to make the correct surgical recommendation.

This article explains how we developed and evaluated the impairment identification and surgical recommendation decision support systems. The decision support system was designed in tight partnership with clinicians in our center, including how the outputs are presented.

## Materials and Methods

### Dataset for Impairment Modeling

#### Data

We collated 3DGA records from the Hugh Williamson Gait Analysis Laboratory (HWGAL) with the following inclusion criteria:

(1) A diagnosis of CP as determined by appropriate clinicians, and registration on the state-wide CP Register.
(2) Data collected from 2008 onward (Prior to 2008, a set of identified impairments was not a mandatory reporting requirement).
(3) 3DGA data must contain at least one barefoot, unassisted walk. No other condition (e.g., orthosis) was included. Typical data collection includes six walking trials with at least three with kinetics data, however, sometimes only the most representative walking trial was uploaded to the report. Representative trials were chosen visually before 2015 and computationally after 2015 (Sangeux and Polak, 2015).
(4) Physical Examination data must be available.
(5) The 3DGA report must list a clear set of identified impairments.

This procedure led to 689 3DGA records being used, stemming from 423 children (mean (SD) age: 10 years (2.4 years), range: 2–21 years). However, we modeled each side (left and right) separately, hence doubling the data to 1378 records. We initially attempted modeling on the individual level, but we found the result to be worse than modeling on each side. We suspect the main reason is that we do not have enough data points to support accurate estimation of many predictors, so when we model both sides together, which effectively doubles the number of predictors, the final predictive accuracy drops. This has been observed previously (Trunk, 1979).

#### Predictors

The predictors that we used can be grouped into two sets: kinematics and physical examination data.

**FIGURE 1:**
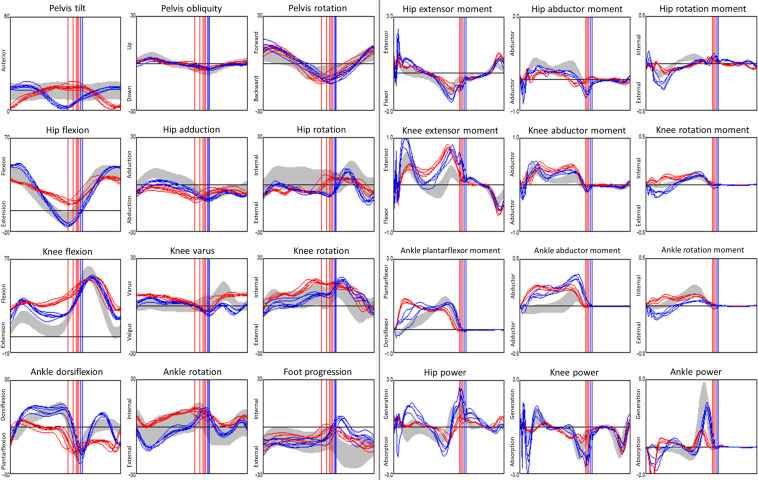
Example of a dataset from gait analysis: 24 kinematic (3 columns from the left, angles in °) and kinetic (3 columns from the right, moments are internal and in N.m/kg bodyweight, powers are in W/kg bodyweight) graphs for a child with CP. Kinematics and kinetics data are plotted along time *t*, in % of the gait cycle. Data from 5 walks were superimposed, the left limb is in red, the right limb in blue, data from typically developed children displayed as a gray band. Vertical lines denote the timing of the ipsilateral foot off.

The kinematics were collected using the following protocol. The children were equipped with the Plug-in-Gait marker set (Vicon, Oxford Metrics Group) by registered physiotherapists. During the 3DGA session, children walked barefoot at their self-selected speed and chose their cadence freely. The three-dimensional marker trajectories were obtained using Vicon motion capture systems including 10 cameras (Oxford Metrics Group, United Kingdom) recording at 100 Hz. The foot strike events, and ground reaction force were captured from 2 (before 2009) to 6 (after 2009) AMTI force plates (AMTI Inc, United States) embedded in the floor. Force plate signals were sampled at 2000 Hz. Lower limb kinematics and kinetics were calculated with Plug-in-Gait in Nexus software (VICON, Oxford Metrics Group) after filtering marker trajectories (Woltring, 1986). We used kinematic data from the lower limbs only namely: Pelvis (sagittal, coronal, transverse), Hip (sagittal, coronal, transverse), Knee (sagittal, coronal, transverse), Ankle (sagittal, transverse), and Foot Progression angle (transverse). All kinematics and kinetics data were normalized to the gait cycle, each curve was described from 101 points, one every % of the gait cycle (Figure 1).

Physical examination data was collected using standard protocols published elsewhere (Keenan et al., 2004; Thomason et al., 2014). Not all physical examination measurements were collected for all children due to difficulty with compliance or physical ability. In our model, we excluded any predictors that were not collected for at least 90% of the children.

Table 1 lists the physical examination predictors that we used in the model.

**TABLE 1:** Physical examination measurements which were used as predictors in the impairment model.

Category	Measurements
Anthropometric	Age, Height, Weight
Strength	Knee extensors, Quadriceps lag, Abdominals, Knee flexors, Hip extensors, Hip abductors, Dorsiflexors, Plantarflexors, Invertors, Hip flexors
ROM/Spasticity (Tardieu fast)	True popliteal angle, Popliteal angle, Dynamic popliteal angle (fast), Dorsiflexion (knee flexed), Dorsiflexion (knee extended), Dynamic dorsiflexion (fast), Hip abduction (knee extended), Hip extension, Duncan-Ely (slow), Duncan-Ely (fast), Hip Internal rotation, Hip external rotation, Selective Motor Control at the ankle
Bone	Femoral anteversion (trochanteric prominence test), Tibial torsion (Bimalleolar axis), Thigh heel angle, Foot posture - Midfoot, Forefoot, Hindfoot sagittal, Hindfoot coronal

*Spasticity measurements were according to Tardieu as the angle of arrest under fast passive range of movement (ROM).*

#### Impairments

We modeled frequently occurring impairments, with impairments that were listed with at least 100 occurrences. Impairments were extracted, tabulated, and added to the database from the text available in the clinical reports. In 2007, clinicians at our center agreed to follow a template to report clinical interpretation of gait analysis. The reporting policy was in line with the concept of impairment focused interpretation which was first described by Baker (2013). Specifically, the impairments listed in the reports were those deemed to impact gait function by the clinician who conducted the 3DGA and completed the interpretation.

Table 2 lists such impairments.

**TABLE 2:** List of impairments with at least 100 occurrences and number of occurrences.

Impairments	Number of occurrences
Hamstring spasticity	497
Gastrocnemius spasticity	434
Increased femoral neck anteversion	383
Soleus spasticity	358
Gastrocnemius contracture	342
Increased external tibial torsion	338
Rectus femoris spasticity	243
Soleus contracture	237
Knee fixed flexion deformity	177
Gluteal weakness	129
Soleus weakness	128
Hip fixed flexion deformity	125
Hamstring contracture	117
Gastrocnemius weakness	107

### Dataset for Surgical Recommendations

#### Data

The inclusion criteria for data used for building the dataset for surgery modeling was the same as for impairment modeling, (1 to 4 listed above) but without the requirement for an impairment list and the addition of:

(6) The child must have undergone surgery within 2 years of the recommendations from the 3DGA report.

This led to 384 3DGA analysis records, stemming from 309 children being included. Again, we modeled each side separately which then doubled the data record number to 618.

#### Predictors

We went through a similar procedure of removing measurements that were not collected for at least 90% of the children. Table 3 lists the physical examination predictors that we used in this model.

**TABLE 3:** Physical examination measurements used for predicting surgical recommendation in the model.

Category	Measurements
Anthropometric	Age, Height, Weight
Strength	Nil
ROM/Spasticity	Hip abduction (knee extended), Dorsiflexion (knee extended), Duncan-Ely (fast), True popliteal angle, Popliteal angle, Hip internal rotation, Hip external rotation
Bone	Tibial torsion (Bimalleolar axis)

#### Surgical Procedures

Table 4 lists the surgical procedures included in the model, each of which were conducted at least 100 times.

**TABLE 4:** Surgical procedures conducted at least 100 times.

Surgeries	Number of times conducted
Femoral derotation osteotomy	159
Semitendinosus transfer	143
Gastrocnemius lengthening (Strayer)	142
Adductor longus lengthening	128

### Modeling

#### Kinematic Feature Extraction

Forty-nine kinematic binary features, all of the form “has X or has not X,” were extracted from the raw kinematic curves. We derived the feature definitions by first using a published DELPHI consensus study (Nieuwenhuys et al., 2016) as a starting point, and then we conducted our own discussion session with clinicians to fine tune the features into the final form as shown in Table 5.

**TABLE 5:** Features extracted from raw kinematic curves.

Structure	Plane	Features	Definition
**Pelvis**	Sagittal	Increased ROM (double bump)	• ROM > 2 SD of typical ROM, and
			• period is 2, and • difference between L&R is <0.25 ROM, and
			• Correlation with our reference double bump curves >0.8.
		Decreased Pelvic Tilt	• Mean angle < 1 SD of typical mean.
		Decreased Pelvic Tilt + Increased ROM	• Decreased Pelvic Tilt, and
			• ROM > 2 SD of typical ROM.
		Increased Pelvic Tilt	• Mean angle > 1 SD from typical mean.
		Increased Pelvic Tilt + Increased ROM	• Increased Pelvic Tilt, and
			• ROM > 2 SD of typical ROM.
		Unilateral Bump	• ROM > 2 SD of typical ROM, and
			• Not double bump.
	Coronal	Increased Pelvic ROM	• ROM > 2 SD of typical ROM
		Pelvic Elevation/Depression	• Mean difference between L&R > 1 SD of typical difference.
	Transverse	Increased Pelvic Rotation ROM	• ROM > 2 SD of typical ROM
		Pelvic Pro / Retraction	• Mean difference between L&R > 1 SD of typical difference.
		Reversed ROM	• Correlation with reference reversed ROM curves >0.8.
**Hip**	Sagittal	Decreased Hip Flexion at Initial Contact	• Angle at *t* = 0 < 2 SD of typical angle.
		Hip Extension Deficit	• Mean angle in stance > 1 SD of typical angle, and
			• ROM < 2 SD of typical ROM.
		Hip Hyper-Flexion	• Mean angle in stance within 1 SD of typical range, and
			• Peak angle in swing > 2 SD of typical peak.
		Increased Hip extension at Mid Stance	• Mean angle at *t*∈[20,45] < 1 SD of typical mean.
		Increased Hip Flexion	• Mean angle in stance > 1 SD of typical mean, and
			• All angles > 0.
		Increased Hip Flexion + Decreased ROM	• Increased Hip Flexion, and • ROM < 2 SD of typical ROM.
	Coronal	Excessive Hip Abduction	• Mean angle in stance < 1 SD of typical mean, and
			• Mean angle in swing < 1 SD of typical mean.
		Excessive Hip Abduction in Swing	• Mean angle in swing < 1 SD of typical mean.
		Excessive Hip Adduction	• Mean angle in stance > 1 SD of typical mean, and
			• Mean angle in swing > 1 SD of typical mean.
		Hip Adduction in Stance	• Mean angle in stance > 1 SD of typical mean.
	Transverse	Hip External Rotation	• Mean angle < 1 SD of typical mean.
		Hip Internal Rotation	• Mean angle > 1 SD of typical mean.
		Increased Hip Internal Rotation at Late Stance	• mean angle in *t*∈[40,60] > 1 SD of typical mean, and
			• peak occurs before *t* = 70, and
			• no pit in *t*∈[20,80]
**Knee**	Sagittal	Reduced Flexion at Loading	• Mean angle in *t*∈[0,20] < 1 SD of typical mean.
		Decreased Peak Knee Flexion	• Peak in swing < 2 SD of typical peak.
		Delayed + Decreased Peak Knee Flexion	• Peak occurs after *t* = 75, and
			• Decreased Peak Knee Flexion.
		Delayed + Increased Peak Knee Flexion	• Peak occurs after *t* = 75, and • Peak in swing > 2 SD of typical peak
		Delayed Peak Knee Flexion	• Peak occurs after *t* = 75.
		Knee Flexion in Mid Stance	• Mean angle in *t*∈[20,45] > 1 SD of typical mean.
		Knee Hyperextension	• Mean angle in *t*∈[20,45] < 1 SD of typical mean.
		Increased Flexion at Initial Contact	• Angle at *t* = 0 > 2 SD of typical angle.
		Increased flexion at Initial Contact+ Early Knee Extension	• Increased flexion at initial contact, and
			• Pit occurs before *t* = 25, and
			• Difference between angle @ IC and at pit > 10, and
			• Min angle in *t*∈[10,25] < mean+1 SD of typical angle
		Increased Peak Knee Flexion	• Peak angle in swing > 2 SD of typical peak.
**Ankle**	Sagittal	Reduced Dorsiflexion	• Mean angle in *t*∈[0,50] < 1 SD of typical mean.
		Descending 2nd Rocker	• Angle at *t* = 45 minus the angle at *t* = 20 < −5, and • Angle at *t* = 45 < 0
		Dorsiflexion in Swing	• Mean angle in swing > 1 SD of typical mean.
		Foot Drop	• Mean angle in *t*∈[80,100] < 1 SD of typical mean.
		Horizontal 2nd Rocker	• ROM in *t*∈[20,45] < 5, and
			• Absolute slope of the same period <0.1, and
			• Angle at *t* = 45 < 0.
		Increased Dorsiflexion	• Mean angle at *t*∈[20,45] > 1 SD of typical mean.
		Increased Max. Dorsiflexion	• Max angle in stance > 2 SD of typical stance.
		Increased Plantarflexion	• Mean angle in *t*∈[20,45] < 1 SD of typical mean.
		Insufficient Pre-positioning	• Angle at *t* = 100 (final) > 2 SD of typical angle.
		No 1st Rocker	• Angle at *t* = 1 > angle at *t* = 0.
		Short 2nd Rocker	• Peak exists in *t*∈[0,20], and
			• Slope in *t*∈[20,45] < 0, and
			• Correlation with reference short 2nd rocker curves >0.8.
	Transverse	Ankle Internal Rotation	• Mean angle in stance > 1 SD of typical angle.
**Foot Progression**	Transverse	External Foot Progression (Wave) in Swing only	• ROM in swing > 2 SD of typical ROM, and
			• Correlation with referenced External foot progression curve >0.8.
		In-toe	• Mean angle in stance > 1 SD of typical mean.
		Out-toe	• Mean angle in stance < 1 SD of typical mean.

*ROM, Range of motion; SD, Standard Deviation; R, Right; L, Left.*

Computationally, these features are detected by applying some function to both the curve under consideration and a set of standard kinematic curves measured from typically developing children (Pinzone et al., 2014; Sangeux et al., 2016), and then making some comparison using means and standard deviation (SD). For example, the feature “Increased Hip extension at Mid Stance” has a definition “Mean angle at *t*∈[20,45] < 1 SD of typical mean.” This translates to a four-step procedure:

(1) Calculate the mean angle for the curve under consideration at *t*∈[20,45].
(2) Calculate the same for each individual curve in the reference (typically developed) dataset.
(3) Calculate the mean and SD of the mean angles calculated in step 2.
(4) If the statistics calculated in step 1 is less than the mean minus 1 SD (as calculated in step 3), then the feature is deemed present in the curve under consideration.

The R code to detect the default kinematic features and create custom-designed feature detectors is available here: https://github.com/Morgan435/gaitFeature/.

Most features in Table 5 are defined over the entire gait cycle or a sub-phase of gait, e.g., stance or swing phase, or at *t* = 0. Markedly different walking speeds between the mean of the typically developing reference dataset and that of the subject under consideration may lead to some time-shift when the features are considered over a specific time period, e.g., *t*∈[20,45] as above. In this case, it is possible to add a pre-processing step that apply dynamic time warping to the curves before features extraction. We did not deem necessary to include such a pre-processing step in this instance.

#### Missing Data Imputation

Missing data were imputed just prior to the model training step. Continuous variables were imputed by their median, and categorical ones were imputed by the most frequently appearing category. We acknowledge that such a simple imputation scheme imposed certain assumptions to the data missing mechanism, such as missing at random, which is unlikely true in clinical practice. But we did check that variables with high missing data did not rank highly in the variable importance matrix.

#### Models

There are many different machine learning algorithms that could be used. We have trained many different models (e.g., support vector machine, linear discriminant model, partial least square, naïve bayes), however, here we report the models with the best overall balance between ease-of-use, computation speed, and predictive performance.

For each impairment / surgical procedure, a standard random forest (Breiman, 2001), a stratified random forest (Kuhn, 2008), and a regularized logistic regression (with elastic net penalty, a.k.a. “glmnet” (Friedman et al., 2010) were fitted. The stratified random forest is like the standard one, except that each tree in the forest is trained on a balanced resample. That is, a resample where the number of instances in each class is equal. The reason for using the stratified version is to tackle class imbalance. However, such stratified resamples are essentially down sampling techniques, that is, throwing away instances of the majority class. Therefore, the cost of a balanced training is higher variance. Hence, the other models were still retained as feasible candidates.

The hyper-parameters were tuned according to the weighted Brier score as defined in the next section.

#### Model Evaluation Metric

One way to assess classification models is to see how well they predict the class of new observations. Metrics such as accuracy, precision, sensitivity, specificity are all designed to assess this aspect of the prediction (Altman and Bland, 1994). However, we advocate moving away from predicting the class to predicting the probability of class membership. This is because, first, unlike predicted class, the predicted probabilities also convey the uncertainty of the prediction. Most of us would probably view 51 and 99% chance of something happening very differently, but in the class prediction sense they would both be considered the same positive prediction, so the uncertainty is lost. Secondly, unlike many artificial intelligence application (e.g., hand-written postcode recognition by the post office), the machine (model) in our clinical setting does not actually have to make any decision, as this responsibility lies with the clinician. Therefore, predicting class membership is in a sense one step more than what is required.

Hence, we decided to use the Brier score (Brier, 1950) as our model evaluation metric. The Brier score is defined as $\sum_{i}{\left(\hat{P_{i}}-O_{i}\right)}^{2}/N$, where $\hat{P_{i}}$ denotes the predicted probability that child *i* had the impairment / surgery, *O*_*i*_ is 1 if the child *i* had the impairment / surgery and 0 otherwise, and *N* is the total number of children. Therefore, Brier score is the mean-squared-error equivalent for binary classification. Another advantage of using Brier score is that it is a proper scoring metric (Harrell, 2015), meaning its expectation is minimized by the true, unknown, probability.

The weighted Brier score is the weighted version where each term in the summation is weighted by the reciprocal of their true class frequency. The reason we used the weighted Brier score is to tackle the issue of class imbalance. By setting the weight to be the reciprocal of the class frequency, error in the minority class would be penalized more heavily, thus forcing us to choose a model that has a more balanced performance in both classes.

A null model which predicts a constant 50% regardless of predictors will achieve a Brier score (weighted or not) of 0.25. Therefore, any admissible model should achieve a score of less than 0.25.

### Training and Validation

Training and validation were done using 25 out-of-bag bootstrap resample. That is, the entire dataset was used to recreate 25 bootstrap resamples, and the models were trained on each resample, and validated on the portion that did not appear in the corresponding training sample.

#### Probability Calibration

Because the predicted probability is our primary output, we carried out a further step of calibrating the prediction probability, using an isotonic regression. The procedure is as followed:

(1) From the previous steps (i.e., model training) collect the prediction made on the out-of-bag (i.e., validation) data, treat this as our new training dataset.
(2) Train an isotonic regression using the predicted probability as predictor, and the observed class as response variable. Such training is also done using the out-of-bag bootstrap resampling scheme.
(3) Using the predicted probability from the trained isotonic regression model as the final predicted probability that we report to the end user.

Probability calibration can sometimes improve the accuracy of the predicted probability, but sometimes impairs it. To ensure this step is beneficial, we once again computed the weighted Brier score and other secondary evaluation metrics and compare them with those from the uncalibrated models.

#### Explanation

We tried to make the model’s prediction as transparent as possible, reasoning that the more transparent the model is, the more informative, and thus helpful, it will be to the end-user clinician. A clinician may not trust a black box that simply declares a certain child has impairment X without providing reason. But the same clinician will likely find a system which suggests a child might have impairment X with Y% certainty because of Z reason(s) more helpful and credible. The reasons for a decision support tool are at least 2-fold. Firstly, the clinician can compare and combine the model’s confidence with their own. If a clinician already has strong belief that impairment X exists, then a model prediction of 60% confidence is most likely enough to reinforce that belief. On the contrary, if the clinician’s prior belief was “highly unlikely,” then even a 60% model confidence may not sway them in their belief. If, however, the model output is 90% confidence, then it might prompt the clinician to further investigate the issue. Secondly, the clinician has the information to examine the reason underlying the prediction, which empowers them to agree with or overrule the prediction. This makes a transparent and trustworthy computer system that has been the subject of many research papers (Ferri et al., 2002; Allahyari and Lavesson, 2011; Freitas, 2013; Castelvecchi, 2016; Lipton, 2016; Ribeiro et al., 2016).

We explain our prediction by firstly, outputting the predicted probability instead of predicted class. Secondly, we display the measurement values of the (up to) 5 most important predictors, where the importance of the predictors is judged by the trained models. In a typical random forest model, the importance metric is related to how much node impurity is reduced by a split on that variable, whereas in a regularized regression, it is related to the absolute magnitude of the coefficients. We say “up to” 5 because for some impairments (e.g., hip fixed flexion deformity), most of the result is explained by less than five predictors (e.g., hip extension ROM). In these cases, it is only meaningful to retain the predictors that have enough weight. Thirdly, we constructed a partial dependence plot, which shows how the models react to changes in each individual predictor, while holding all other predictors constant (or averaging over them). If our models were simply linear regression, these plots would simply be a straight line with the slope being to the estimated coefficient. But for algorithms such as random forest, the dependency can be non-linear.

## Results

### Impairment Diagnosis Model

Table 6 reports the weighted Brier score of the various impairment diagnosis models on the validation sample. Recall that a Brier score can be loosely thought of as the mean squared error of the predicted probability (thus lower equals better), and a null model has a score of 0.25. For the calibrated random forest, we have also reported the mis-classification rate, sensitivity, and specificity. We have placed the models which fail to give a weighted Brier score or less than 0.25 at the bottom of the Table. Overall, the calibrated (standard) random forest is usually the best model in terms of having the lowest weighted Brier score. However, for all the muscle weakness impairments, all models failed to be better than the null model in the weighted Brier score.

**TABLE 6:** Weighted (out-of-bag/validation) Brier score for impairment diagnosis model, and the associated important variables.

Impairments	Glmnet		Stratified R.F.		Random Forest		Important Predictors
	Raw	Calibrated	Raw	Calibrated	Raw	Calibrated	By Random Forest
Hamstring Spasticity	0.186	0.187	0.174	0.169	0.172	0.166 Mis:0.21 Sens:0.71 Spec:0.83	1. Dynamic Popliteal Angle 2. Height 3. Weight 4. Popliteal Angle 5. True Popliteal Angle
Gastrocnemius Spasticity	0.216	0.219	0.198	0.200	0.196	0.195 Mis:0.23 Sens:0.68 Spec:0.80	1. Dynamic Dorsiflexion 2. Height 3. Weight 4. Increased Plantarflexion 5. Dorsiflexion (Knee Flexed)
Increased Femoral-Neck Anteversion	0.176	0.174	0.165	0.168	0.163	0.163 Mis:0.17 Sens:0.70 Spec:0.89	1. Hip Internal Rotation 2. Anteversion 3. Feature: Hip Internal Rotation 4. Weight 5. Hip External Rotation
Soleus Spasticity	0.245	0.246	0.223	0.226	0.220	0.218 Mis:0.22 Sens:0.60 Spec:0.82	1. Dynamic Dorsiflexion 2. Height 3. Weight 4. Dorsiflexion (Knee Extended) 5. Feature: Increased Plantarflexion
Gastrocnemius Contracture	0.140	0.152	0.132	0.147	0.132	0.144 Mis:0.13 Sens:0.74 Spec:0.91	1. Dorsiflexion (Knee Extended) 2. Dorsiflexion (Knee Flexed) 3. Dynamic Dorsiflexion
Increased External Tibial Torsion	0.194	0.197	0.183	0.188	0.183	0.181 Mis:0.17 Sens:0.71 Spec:0.86	1. Tibial Torsion 2. Thigh Heel Angle
Rectus Femoris Spasticity	0.192	0.194	0.176	0.179	0.166	0.158 Mis:0.09 Sens:0.83 Spec:0.92	1. Feature: Increased ROM (Pelvis) 2. Duncan-Ely (Fast) 3. Duncan-Ely (Slow) 4. Weight 5. Height
Soleus Contracture	0.165	0.179	0.158	0.174	0.161	0.174 Mis:0.11 Sens:0.71 Spec:0.92	1. Dorsiflexion (Knee Flexed) 2. Dorsiflexion (Knee Extended) 3. Dynamic Dorsiflexion
Hip Fixed Flexion Deformity	0.184	0.191	0.148	0.186	0.148	0.172 Mis:0.07 Sens:0.71 Spec:0.95	1. Hip Extension ROM

**Models Which Are Not Better Than The Null Model (In Terms Of Weighted Brier Score)**							

Hamstring Contracture	0.296	0.310	0.262	0.291	0.249	0.245 Mis:0.08 Sens:0.62 Spec:0.94	1. Popliteal Angle 2. Dynamic Popliteal Angle 3. Weight 4. True Popliteal Angle 5. Height
Knee Fixed Flexion Deformity	0.277	0.292	0.276	0.298	0.276	0.281 Mis:0.12 Sens:0.64 Spec:0.90	1. Quadriceps Strength 2. True Popliteal Angle 3. Hip Abduction (Knee Extended) 4. Hip External Rotation 5. Popliteal Angle
Gluteal Weakness	0.307	0.324	0.310	0.333	0.302	0.280 Mis:0.08 Sens:0.71 Spec:0.93	1. Dynamic Popliteal 2. Height 3. Weight 4. Popliteal Angle 5. True Popliteal Angle
Soleus Weakness	0.303	0.315	0.304	0.330	0.297	0.272 Mis:0.08 Sens:0.59 Spec:0.94	1. True Popliteal Angle 2. Hip Abduction (Knee Extended) 3. Hip Extension ROM 4. Weight 5. Anteversion
Gastrocnemius Weakness	0.331	0.342	0.335	0.361	0.325	0.298 Mis:0.07 Sens:0.59 Spec:0.94	1. True Popliteal Angle 2. Popliteal Angle 3. Dorsiflexion (Knee Extended) 4. Hip Extension ROM 5. Hip Abduction (Knee Extended)

*For calibrated random forest, the mis-classification rate, sensitivity, and specificity are also reported. RF, random forest; ROM, Range of motion; Mis, mis-classification rate; Sen, sensitivity; and Spec, specificity.*

**FIGURE 2:**
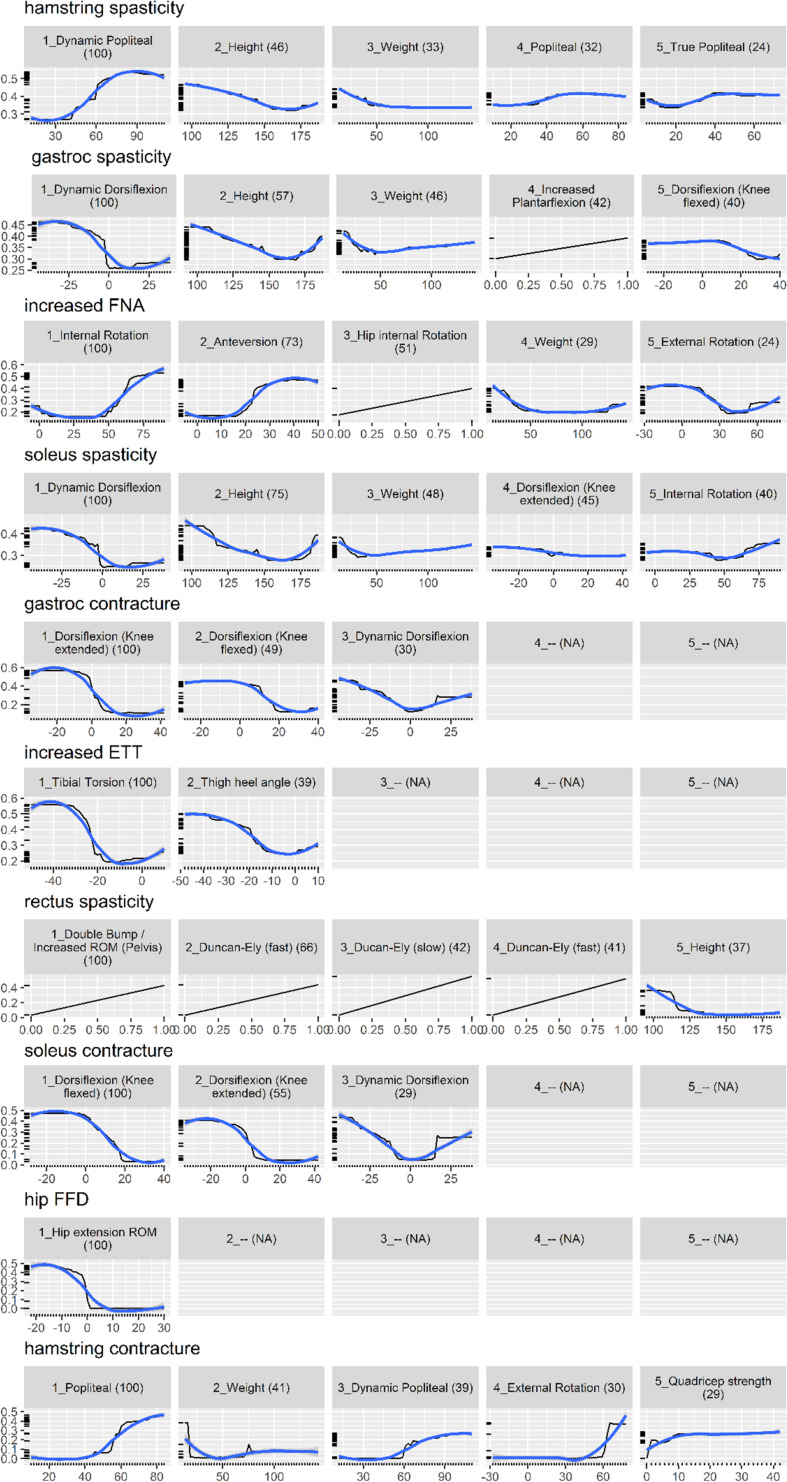
Partial dependence plot for the impairment prediction model (black line, 30). The vertical axis is the predicted probability (between 0 and 1) that the impairment is present, on a log scale. The horizontal axis is the measurement for the predictor. The (blue) smooth line is added for visual aid. For some impairments, there are less than five important predictors, resulting in blank panels. Finally, the number in parenthesis indicates the importance of that predictor, relative to the most important predictor (so it always starts from 100 and decreases).

Figure 2 shows the partial dependence plot for the impairment model (Friedman, 2001). The vertical axis is predicted probability that the impairment is present on the log scale. Some plots only show a straight line, as the predictors are binary (most likely one of the kinematic feature variables).

### Surgical Recommendation Model

Table 7 presents the weighted Brier score for the surgical recommendation prediction model. Similarly, for the calibrated random forest, the mis-classification rate, sensitivity, and specificity are also provided. Overall, the two flavors of random forest perform very similarly, with the standard version being marginally better.

**TABLE 7:** Weighted Brier score surgery recommendation models.

Surgery	Glmnet		Stratified R.F.		Random Forest		Important predictors
	raw	calibrated	Raw	calibrated	raw	calibrated	by random forest
Femur derotation osteotomy	0.173	0.172	0.163	0.163	**0.163**	0.164 mis:0.22 sens:0.78 spec:0.78	1. Internal Rotation 2. Feature: Hip internal Rotation 3. External Rotation 4. Weight 5. Abduction (knee extended)
Gastrocnemius lengthening (Strayer)	0.226	0.227	0.202	0.207	**0.201**	0.204 mis:0.24 sens:0.67 spec:0.80	1. Dorsiflexion (Knee extended) 2. Height 3. Internal Rotation 4. Abduction (knee extended) 5. Dorsiflexion (Knee flexed)
Semitendinosus transfer	0.196	0.197	0.177	0.176	0.175	**0.172 mis:0.20 sens:0.73 spec:0.83**	1. Popliteal 2. Abduction (knee extended) 3. True Popliteal 4. Height 5. Weight
Adductor longus lengthening	0.240	0.241	**0.223**	0.226	0.226	0.223 mis:0.24 sens:0.64 spec:0.79	1. Abduction (knee extended) 2. Internal Rotation 3. External Rotation 4. Weight 5. Height
Tibial derotation osteotomy	0.233	0.235	0.209	0.224	**0.208**	0.215 mis:0.14 sens:0.67 spec:0.89	1. Tibial Torsion 2. Dorsiflexion (Knee flexed)
Rectus transfer	0.255	0.257	0.226	0.250	0.220	**0.219 mis:0.13 sens:0.75 spec:0.88**	1. Popliteal 2. Tibial Torsion 3. Weight 4. Abduction (knee extended) 5. Height

*For the calibrated random forest, mis-classification rate, sensitivity, and specificity are also provided. The right most column lists the top five important predictors as judged by the random forest. RF, random forest; ROM, Range of motion; Mis, mis-classification rate; Sen, sensitivity; and Spec, specificity.*

**FIGURE 3:**
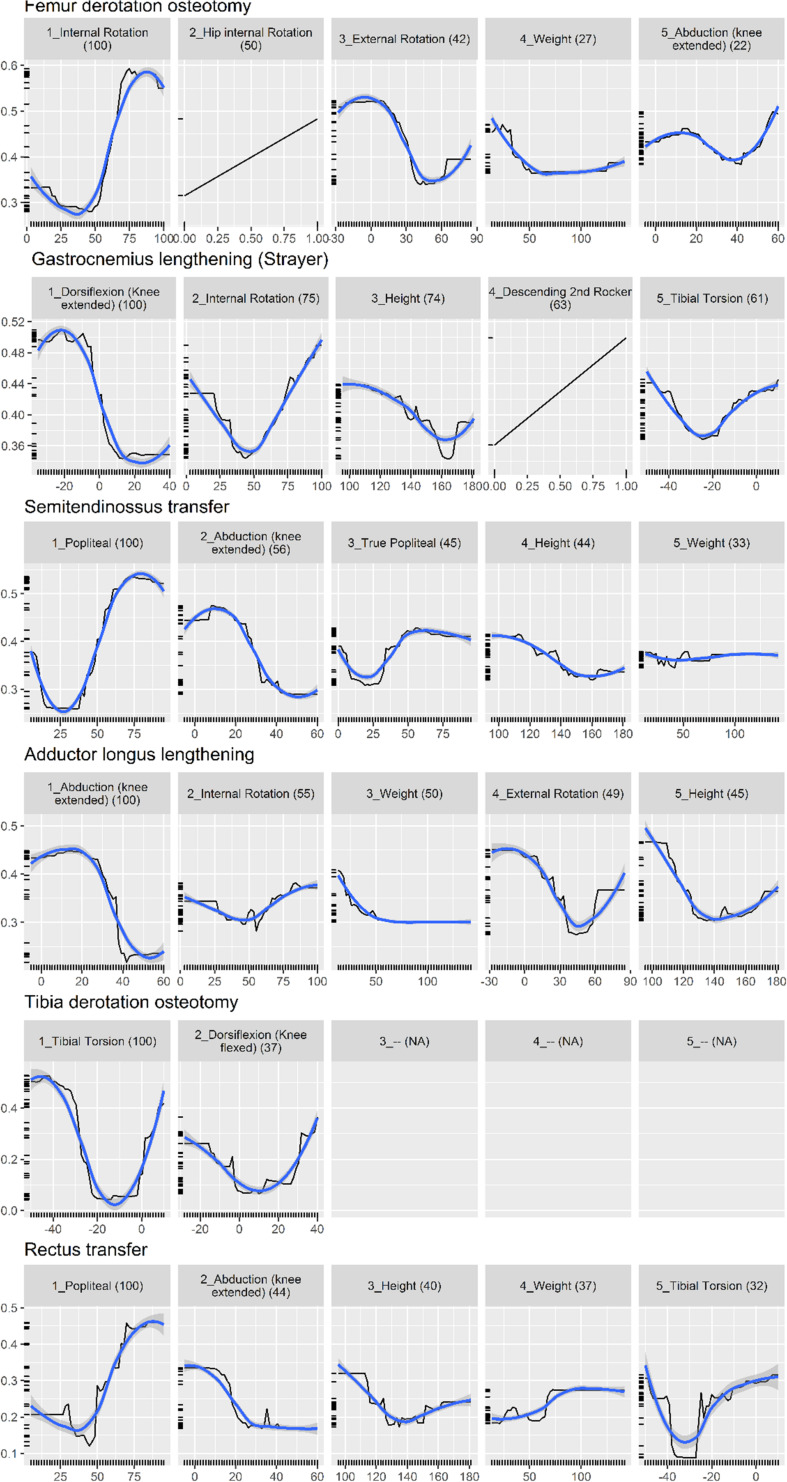
Partial dependence plot for surgery prediction model (black line, 30). The vertical axis shows the predicted probability (between 0 and 1) that the surgery is needed, on a log scale. The horizontal axis is the measurement for the predictor. The (blue) smooth line is added for visual aid. For some surgeries, there are less than five important predictors, resulting in blank panels. Finally, the number in parenthesis indicates the importance of that predictor, relative to the most important predictor (so it always starts from 100 and decreases).

Figure 3 shows the partial dependence plot for the surgery model.

### System Output

**FIGURE 4:**
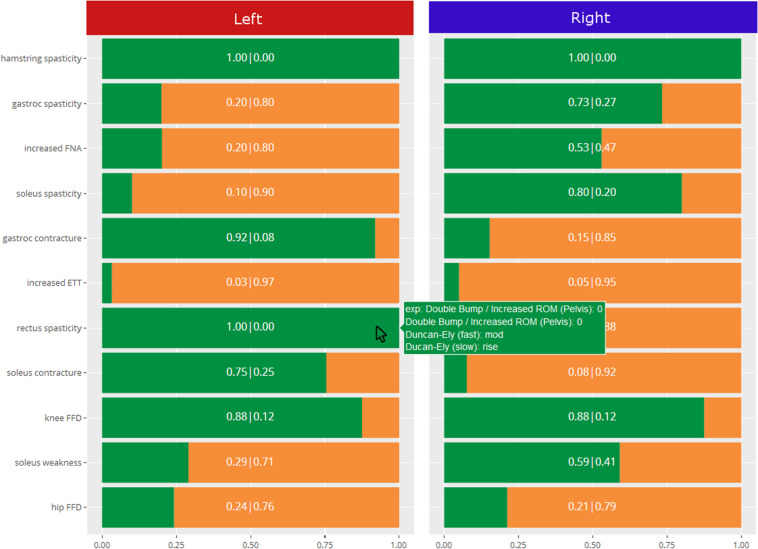
Sample output from the decision support system for a specific patient.

In order to give readers a sense of the decision support system, Figure 4 shows the output from the impairment model. The green reflects the predicted probability that the impairment is present, and the orange is the complement to that. As can be seen, the predicted probability rather than predicted class is the primary output, and the explanation behind the prediction is reported.

## Discussion

We have developed two sets of models to facilitate identification of musculoskeletal impairments and surgical recommendations. These models are meant to reproduce the essence of past clinical reasoning to new data. The models were trained to associate kinematic features and physical examination data with the list of impairments from the clinical interpretation report and the list of surgical treatments. Our approach is original because it proposes an intermediate step, between the use of computerized tools using explicit knowledge, e.g., (Wagner et al., 2019), and data-driven approach to determine which treatment should be recommended (or should a particular treatment be recommended) by maximizing the likelihood of good outcome measures, e.g., (Ries et al., 2014). The primary output was the calibrated confidence that a certain impairment is present/absent, together with the values of the five most important predictors. Partial dependence plots were also supplied to assist understanding of the general reasoning of the models.

Physical examination measurements were the most important predictors for impairments, listed as impacting gait in the interpretation report, and surgical recommendations. The partial dependence plots in Figures 2, 3 provide some indications about the soft threshold values linking certain physical examination measurements with impairments. For many impairments and surgeries, these were the only predictors deemed important by the models. Of note, only a reduced set of physical examination measurements could be used for surgical recommendation models because of missing data. Only three impairments: increased femoral neck anteversion (feature: increased hip internal rotation), soleus spasticity (feature: increased plantarflexion) and rectus femoris spasticity (feature: double bump) included a kinematic feature as an important predictor. For models to predict surgical recommendations, only the femoral derotation osteotomy surgery included a kinematic feature (increased hip internal rotation) as an important predictor.

These results were unexpected and somewhat contrary to our opinion about the influence of lower limb kinematics on clinical decision making. One explanation may be that kinematic data, as curves, are difficult to include in a predictive statistical model because of their high dimensionality (101 points times 15 curves). We proposed a dimension reduction process that summarizes the kinematics curves into a set of kinematic features. We may have lost some important information during this process. However, we initially also treated the curves directly and obtained worse weighted Brier scores for the various models (Chia et al., 2017). Another explanation may be that kinematic data are essential to confirm the impact of a physical examination measurement on the gait pattern, but that decisions ultimately hinge upon physical examination measurements.

There are arguably more advanced methods to achieve transparent predictions than those we implemented. For example, one approach may be to use a comprehensible model, such as a (single) decision tree to approximate the behavior of the models, either globally (Domingos, 1998; Martens et al., 2007) or locally (Ribeiro et al., 2016). The advantage of this approach is that the full decision pathway is explained, instead of our current approach which simply returns the values of some predictors which are deemed important. However, the problem of all approximation is that it loses predictive power. In addition, the fact that we calibrate our model further complicates the process of explaining the prediction. Another area of challenge is the class imbalance problem. The class imbalance is both in terms of occurrence, as well as cost of error. For imbalance in occurrence, we tried to tackle them with stratified random forest, but the overall performance was not better than the standard version. For imbalance in cost, we could have trained cost-sensitive models, which penalize error in both classes differently, and according to a pre-specified cost. Eliciting such cost structure is non-trivial (e.g., what is the cost of missing an impairment or recommending an unnecessary surgery?) but would be a worthwhile pursuit.

Our predictive models seek to imitate the past behavior of the clinicians at our center. The limitation of this approach is that past errors will also be learnt by the model, which is why it is important to constantly refresh and update the model with new data, which will dilute the influence of past decisions.

Another limitation is that these predictive models are, by intent and because the models were trained on data and clinical reasoning from a single center, not generalizable to other centers. However, there is also a benefit to this approach. In our gait laboratory, data are discussed at a team reporting meeting where recommendations are made. The models capture the collective, therefore representative, reasoning of the clinical team. If each center trains their own set of models, the comparison of these models would highlight the similarities and differences between the clinical practices objectively. For example, it would be possible to perform virtual visit(s), whereby the same clinical and gait analysis data would be fed to the models from different centers, likely leading to different conclusions (Noonan et al., 2003). This would initiate fruitful discussion about the rationale behind the differences in reasoning and would allow the comparison of outcomes drawn from independent samples. This could be a key element in making progress toward the search for evidence-based optimal treatment recommendations.

## Conclusion

We presented a decision-support system able to propose a list of impairments and surgical recommendations based on past decisions and gait analysis datasets. Machine learning models were trained and validated to predict the probability that clinicians, experienced in the interpretation of gait analysis data in children with CP, recommend an impairment or a surgical procedure. The random forest algorithm provided the best evaluation metrics (weighted Brier score) in most cases. Overall, the models achieved a weighted Brier score lower than 0.20, and sensitivity and specificity greater than 0.70 and 0.80, respectively. Once trained, these models collectively store the relationship between clinical decisions and gait data at our centre. The implementation of similar models in other center would facilitate objective comparison of clinical decision making, or “philosophy,” between centers.

## Data Availability Statement

The datasets generated for this study will not be made publicly available. The dataset is composed of patient sensitive information.

## Ethics Statement

The studies involving human participants were reviewed and approved by The Royal Children’s Hospital Melbourne Ethics Committee. Written informed consent from the participants’ legal guardian/next of kin was not required to participate in this study in accordance with the national legislation and the institutional requirements.

## Author Contributions

KC, IF, and MS contributed to the conception of the study and development of the machine learning models and infrastructure. KC performed the statistical analyses. KC and MS wrote the first draft of the manuscript, with sections contributed by PT and HG. All authors contributed to manuscript revision, read, and approved the submitted version.

## Conflict of Interest

The authors declare that the research was conducted in the absence of any commercial or financial relationships that could be construed as a potential conflict of interest.
